# Dietary tryptophan ameliorates metalaxyl-induced colitis by restoring microbial tryptophan metabolism and aryl hydrocarbon receptor activation

**DOI:** 10.3389/fnut.2026.1930000

**Published:** 2026-08-20

**Authors:** Wei Sun, Yu Wang, Xin Zhang, Yifan Yue, Xiaoxuan Sun, Jiyan Miao, Shihang Han, Zhiqiang Zhou, Xiuguo Wang, Wentao Zhu

**Affiliations:** 1 State Tobacco Monopoly Administration (China National Tobacco Corporation), Key Laboratory of Tobacco Pest Monitoring Controlling & Integrated Management, Tobacco Research Institute of Chinese Academy of Agricultural Sciences, Qingdao, China; 2 Innovation Center of Pesticide Research, Department of Applied Chemistry, College of Science, China Agricultural University, Beijing, China

**Keywords:** aryl hydrocarbon receptor, gut microbiota, inflammatory bowel disease, metalaxyl, tryptophan metabolism

## Abstract

**Introduction:**

The extensive production of environmental pollutants has heightened susceptibility to intestinal disorders, potentially through alterations in the gut microbiota. However, the precise role of the gut microbiota in environmental pollutant-induced inflammation remains incompletely understood.

**Methods:**

In this study, wild-type and IL-10^−/−^ mice were employed to simulate the responses of healthy individuals and those genetically predisposed to inflammatory bowel disease (IBD) to environmental toxicants. Mice were exposed to metalaxyl, and an integrated multi-omics approach combining microbiome and metabolome profiling with machine learning was conducted to investigate the underlying mechanisms. Additionally, dietary tryptophan supplementation was administered to evaluate its protective effects.

**Results:**

Metalaxyl exposure induced low-grade colonic inflammation in wild-type mice and triggered severe colitis in IBD-susceptible mice. Multi-omics analysis revealed that metalaxyl significantly disrupted gut microbial composition, reduced the synthesis of endogenous tryptophan-derived metabolites, and inhibited aryl hydrocarbon receptor (AhR) signaling, ultimately initiating intestinal barrier dysfunction and inflammatory cascades. Notably, dietary tryptophan restored gut tryptophan metabolite levels, strengthened AhR signaling, mitigated intestinal inflammation, and repaired barrier defects.

**Discussion:**

This study identifies the gut microbiota as a central mediator of environmental pollutant-induced IBD and demonstrates that a high-tryptophan diet exerts beneficial effects against environmental colitis via the AhR axis. Furthermore, the stable phenotypes and high reproducibility of this dual-genotype mouse model underscore its utility as an ideal platform for investigating the enterotoxic effects of environmental pollutants.

## Graphical abstract

**Figure:**
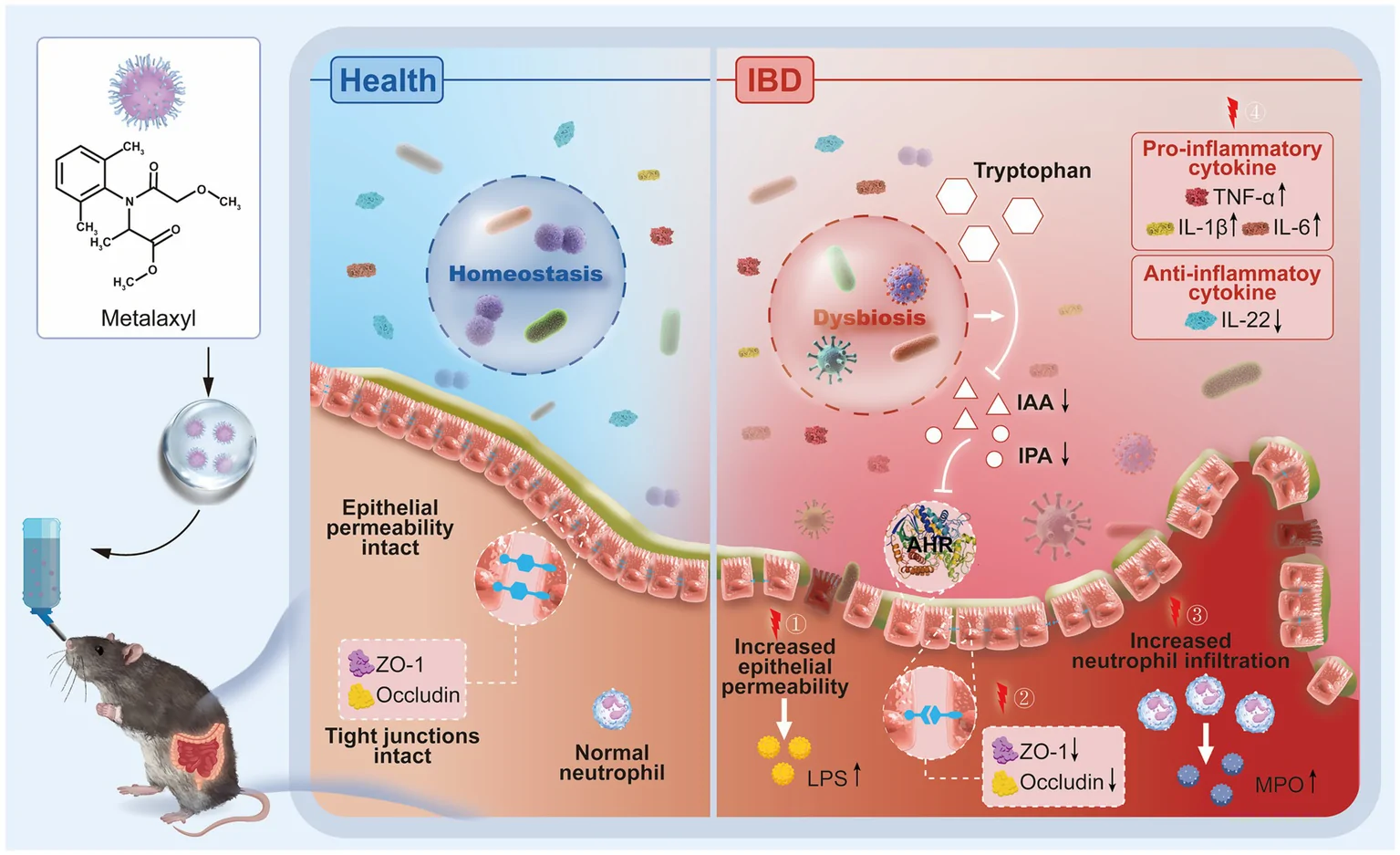

## 1 Introduction

Inflammatory bowel disease (IBD), a chronic intestinal inflammatory disorder, has become a significant burden on global healthcare expenditures (1). Epidemiological data indicate that the prevalence of IBD in high-incidence countries in Europe and North America has surpassed 0.7%, and its incidence is on the rise year by year in emerging industrialized countries in Asia, Africa, and South America (2). A growing body of studies has demonstrated that exposure to environmental pollutants, including pesticides (3), heavy metals (4, 5), microplastics (6), perfluorinated and polyfluoroalkyl substances (7, 8) constitutes a critical risk factor exacerbating the global prevalence of IBD. Metalaxyl, one of the most commonly used systemic fungicides in current agriculture, is widely applied worldwide to prevent and control plant diseases caused by various pathogens (9). However, due to its extensive application, high water solubility and relatively long half-life, its residues are widely found in environmental matrices such as soil (10, 11), water (12, 13), and air (14). Moreover, metalaxyl residues have been detected in food to varying degrees. For example, a survey in China showed that the detection rate of metalaxyl in 192 samples of bayberries was as high as 97.92%, with an average residue level of 0.07 mg/kg (15). In addition, the residue levels in tomato samples from Nepal and Kenya could reach as high as 19.60 mg/kg and 5.61 mg/kg, respectively (16), far exceeding the specified maximum residue limits. These metalaxyl residues may enter the human body through dietary pathways and potentially pose a threat to healthy intestine.

The gut microbiota is a dense and diverse microbial community that has established a mutualistic symbiotic relationship with the host through co-evolution (17, 18). There is ample evidence that the gut microbiota plays a vital role in regulating intestinal immune homeostasis and maintaining the integrity of the mucosal barrier (19, 20). Notably, as a key interface of “environment–host” interaction, the gut microbiota is highly susceptible to perturbation by environmental pollutants (21). For a long time, researchers have hypothesized that the gut microbiota plays a vital role in the progression of IBD triggered by environmental pollutants including pesticides, yet it remains unclear whether this process is mediated by shifts in microbial community composition.

Small-molecule metabolites derived from the gut microbial community are increasingly recognized as critical mediators governing host physiological functions. These metabolites, as the integrated output of the entire microbial community’s functions, are capable of directly and comprehensively modulating the host’s intestinal homeostasis via specific receptors (22). Among them, tryptophan derivatives, which are a key group of metabolites produced by microbes, can orchestrate immune responses and are involved in regulating tight junctions and mucins through activation of the aryl hydrocarbon receptor (AhR) or the pregnane X receptor (PXR) (23–25). Recent studies have indicated that certain dietary bioactive compounds, such as pectin (26) and polyphenols (27), can modulate the composition of gut microbiota and metabolites, and exhibit therapeutic potential against intestinal inflammation and injury. Nevertheless, the mitigating effects of a high-tryptophan diet on IBD, particularly pollutant-induced IBD, remain to be further elucidated.

Based on this, this study utilized wild-type and IBD-susceptible mouse models, simulated exposure scenarios of metalaxyl, evaluated its effects on gut health of hosts with different genetic backgrounds, and elucidated the specific role and pathogenic mechanism of gut microbiota in enhancing susceptibility to metalaxyl induced colitis through multi-omics joint analysis and machine learning modeling. At the same time, the protective effect of dietary supplementation with tryptophan on IBD induced by environmental pollutants was verified. This study provides direct evidence for the link between exposure to environmental pollutants, gut microbiota, and intestinal inflammation, and emphasizes the important role of intervention strategies with high-tryptophan diets in the treatment of environmental IBD.

## 2 Materials and methods

### 2.1 Chemicals and materials

Metalaxyl (purity 99.0%), neomycin (99.0%), metronidazole (99.0%), gentamicin (97.5%), ampicillin (98.0%), vancomycin (98.5%) were sourced from Shanghai Aladdin (Shanghai, China). 6-formylindolo[3,2-b]carbazole (purity≥95%) was sourced from Sigma-Aldrich (MO, USA). Cyp1a1 polyclonal antibody was sourced from Wuhan Sanying (Wuhan, China).

### 2.2 Animal experiments

Four-week-old male wild-type C57BL/6 mice and *IL-10*^−/−^ C57BL/6 mice were sourced from Beijing Vital River Laboratory Animal Technology Co., Ltd. and Cyagen Biosciences Inc., respectively. The experimental environment was maintained at room temperature 23 ± 2 °C with a 12/12-h light/dark cycle, and sterile water and standard chow (SPF Biotechnology Co., Ltd.) were provided ad libitum to the mice. The experimental design and procedures were carried out in compliance with the guidelines of the Independent Animal Ethics Committee of China Agricultural University (Approval No.: AW91603202-5-2).

#### 2.2.1 Animal experiment 1: intestinal toxicity testing experiment of metalaxyl

For *IL-10*^−/−^ type mice, after 1 week of adaptive feeding, they were randomly divided into two groups (*n* = 6 in each group), one group of mice was orally given deionized water without metalaxyl as the control group (Ctrl group); the other group of mice was orally supplemented with deionized water at a concentration of 0.8 mg/L of metalaxyl as the metalaxyl exposure group (Met group). For wild-type mice, there were also two groups that received deionized water without metalaxyl (Ctrl group) and deionized water supplemented with 0.8 mg/L metalaxyl (L-Met group), respectively. Additionally, another group of mice was administered deionized water supplemented with a higher dose of metalaxyl (8 mg/L) to observe as many adverse effects on wild-type mice as possible, designated as the high-dose metalaxyl treatment group (H-Met group). All exposure solutions were changed every 2 days, and after continuous exposure for 12 weeks, the mice were euthanized using CO_2_ asphyxiation method. The entire colon was excised for measurement and samples of serum, colon, cecal contents, etc. were collected for subsequent analysis. The schematic of the experimental design is provided in the Results section.

#### 2.2.2 Animal experiment 2: fecal microbiota transplantation experiment

Collect fresh feces from each group of mice in animal experiment 1 as donor microbiota, and perform fecal microbiota transplantation according to previous research methods (28). *IL-10*^−/−^ type mice and wild-type mice were, respectively, divided into two groups (*n =* 6 for each group), mice were administered a mixture of antibiotics (0.250 mg/day of neomycin, gentamicin, metronidazole, ampicillin, and 0.125 mg/day of vancomycin) once daily for 12 consecutive days, and then re-colonized 3 days later via daily oral gavage of donor microbiota for 7 days. To consolidate the donor microbiota genotype, the microbiota were additionally administered weekly throughout the experiment. Re-colonized with Ctrl group’s microbiota, assigned as R-Ctrl group; Re-colonized with Met group’s microbiota (the donor microbiota of wild-type mice comes from the H-Met group), assigned as R-Met group. Upon 12 weeks of continuous exposure, the mice were euthanized and samples were collected for further analysis. Preparation of donor microbiota suspension: dilute fresh feces with physiological saline (1:9, w/w), vortex homogenize for 1 min, centrifuge at 500 g for 3 min, and collect the supernatant. The schematic of the experimental design is provided in the Results section.

#### 2.2.3 Animal experiment 3: AhR activators treatment experiment

*IL-10*^−/−^ type mice and wild-type mice were, respectively, divided into four groups (*n =* 6 for each group), mice were provided with free access to deionized water supplemented with or without metalaxyl for 12 weeks, followed by intraperitoneal injection 6-formylindolo[3,2-b] carbazole or carrier reagent (DMSO and normal saline) once every 5 days. The injection dose of 6-formylindolo[3,2-b] carbazole was 1 ug per mouse. The treatment groups are as follows: (1) Ctrl group (oral deionized water and inject carrier reagent); (2) Met group (oral deionized water supplemented with metalaxyl and inject carrier reagent); (3) Met + Ficz group (oral deionized water supplemented with metalaxyl and inject 6-formylindolo[3,2-b] carbazole); (4) Ficz group (oral deionized water and inject 6-formylindolo[3,2-b] carbazole). Upon 12 weeks of continuous exposure, the mice were euthanized and samples were collected. The schematic of the experimental design is provided in the Results section.

#### 2.2.4 Animal experiment 4: high tryptophan diet treatment experiment

*IL-10*^−/−^ type mice and wild-type mice were, respectively, divided into four groups (*n* = 6 for each group), mice were provided with free access to deionized water supplemented with or without metalaxyl for 12 weeks, meanwhile, the mice were fed a regular diet (the tryptophan content was 2.7 g/kg) or a tryptophan-rich diet (the tryptophan content was 10 g/kg). The nutritional components of the diet are listed in Supplementary Table S1. The treatment groups are as follows: (1) Ctrl group (oral deionized water and fed a regular diet); (2) Met group (oral deionized water supplemented with metalaxyl and fed a regular diet); (3) Met + Trp group (oral deionized water supplemented with metalaxyl and fed a tryptophan-rich diet); (4) Trp group (oral deionized water and fed a tryptophan-rich diet). Upon 12 weeks of continuous exposure, the mice were euthanized and samples were collected. The schematic of the experimental design is provided in the Results section.

### 2.3 Biochemical parameters analysis

The activity of myeloperoxidase (MPO) in colon and diamine oxidase (DAO) in serum and the level of lipopolysaccharide (LPS) in serum were measured by using kits according to the manufacturer’s instructions.

### 2.4 Histopathological analysis

The colon of mice was fixed in 4% paraformaldehyde fix solution, dehydrated and embedded in paraffin, stained with hematoxylin eosin (H&E). Then, they were imaged and assessed using the Olympus BX51 imaging system (Olympus Corporation, Japan) and Image-Pro Plus 6.0 software (Media Cybernetics, USA). Samples were evaluated by medical experts blinded to the experimental status. The degree of inflammatory infiltration and histopathological changes in crypt structure were used to evaluate histological scoring.

### 2.5 Quantitative RT-qPCR analysis

The colonic tissue was utilized to extract total RNA using TRIzol reagent. cDNA was then synthesized from the extracted RNA via reverse transcription employing the FastQuant RT Kit. RT-qPCR analysis was performed using the SuperReal PreMix Plus kit on a Bio-Rad CFX 96 PCR system (Hercules, USA). The primers for the target gene were sourced from Bioengineering Co., Ltd. (Shanghai, China); the specific primer sequences are provided in Supplementary Table S2.

### 2.6 Western blotting analysis

Colon tissue was lysed using RIPA lysis buffer to extract total protein, and protein concentration was measured. Then the protein samples were separated and extracted by 10% SDS-PAGE gel, transferred to PVDF membrane, and sealed with 5% skimmed milk at room temperature for 1 h. Subsequently, the membrane was incubated overnight with primary antibody (Cyp1a1 polyclonal antibody, 1:1000) at 4 °C. After washing three times with 1 × PBST, incubate the membrane with the second antibody at room temperature for 60 min. Using *β*-actin as an internal reference protein, followed by analysis using a chemiluminescence imaging system.

### 2.7 16S rRNA sequencing analysis

Microbial DNA extraction and sequencing studies refer to previous reports (29). Briefly, The QIAamp DNA Fecal Mini Kit was used to extract microbial DNA from cecal contents. DNA concentration was measured with a UV spectrophotometer, and DNA quality was assessed via 0.8% agarose gel electrophoresis. The V3-V4 region of 16 s rRNA was targeted for PCR amplification using primers 338F and 806R. PCR products were purified through 2% agarose gel electrophoresis and quantified by fluorescence. Paired-end sequencing of DNA fragments was conducted on the Illumina MiSeq platform (Illumina, San Diego, USA). Statistical analysis was carried out with QIIME software (v2.0, http://www.qiime.org/) and the SILVA database. The original contributions presented in the study are publicly available. This data can be found here: (https://www.ncbi.nlm.nih.gov/sra/PRJNA1509751, accession number: PRJNA1509751).

### 2.8 Metabolomics analysis

The cecum contents were rapidly frozen in liquid nitrogen, and the frozen samples were freeze-dried for 12 h in a vacuum freeze dryer. 1,000 μL of a pre-cooled methanol–water solution (with a ratio of 80:20 methanol to water) containing the internal standard (^13^C-glutamine) was added to 20 mg of freeze-dried material. The mixture was vortexed for 30 s, then sonicated in an ice water bath for 10 min. Next, the sample was incubated at −80 °C for 1 h. Then, the sample underwented centrifugation at 15,000 g at 4 °C for a duration of 10 min. A certain amount of the supernatant was transferred to an injection vial and the internal standard (^13^C-alanine) was added for LC–MS/MS analysis.

Using SCIEX Triple Quad 6500 + LC–MS/MS targeted detection and analysis the pretreated cecal contents. The analysis was conducted using an XBridge Premier BEH Amide Column (2.5 μm, 2.1 mm × 150 mm, 1/pk). Solvent A is composed of 90% water, 10% acetonitrile, 0.2% acetic acid and 5 mM ammonium acetate. Solvent B contains 90% acetonitrile, 10% water, 0.2% acetic acid and 5 mM ammonium acetate. The total gradient elution time is set at 25 min. The detailed metabolites were provided in Supplementary Table S3.

### 2.9 Random forest-based joint analysis of gut microbiota and metabolites

A random forest classification model was constructed using the screened differential genera and differential metabolites as input features. The relative abundance at the genus level was adopted for microbiota data, and the peak areas of metabolites were subjected to log₁₀(*x* + 1) transformation. Three independent models were built separately using microbiota features, metabolite features, and integrated combined features, with the number of decision trees set to 200 and class balance weights applied. Model performance was assessed via leave-one-out cross-validation (LOOCV), with calculations averaged across five replicates using distinct random seeds. The area under the receiver operating characteristic curve (AUC) and accuracy were used to quantify model performance. Permutation tests with 120 rounds of label shuffling combined with 5-fold cross-validation were performed to verify the statistical significance of the established models. Feature importance was quantified based on the Gini coefficient, and the final feature ranking was presented as mean ± standard deviation calculated from 30 repeated model training runs.

### 2.10 Statistical analysis

Independent sample *t*-tests were conducted using SPSS 19.0 to show statistical differences between the control group and the treatment group. Compare the differences between multiple groups using one-way ANOVA followed by Tukey’s *post-hoc* test. In this experiment, *p* < 0.05 was considered statistically significant. For data visualization, GraphPad Prism software was utilized, with all data visually depicted as mean ± standard deviation (mean ± SD).

## 3 Results

### 3.1 Metalaxyl exposure caused colonic inflammation in mice

**Figure 1:**
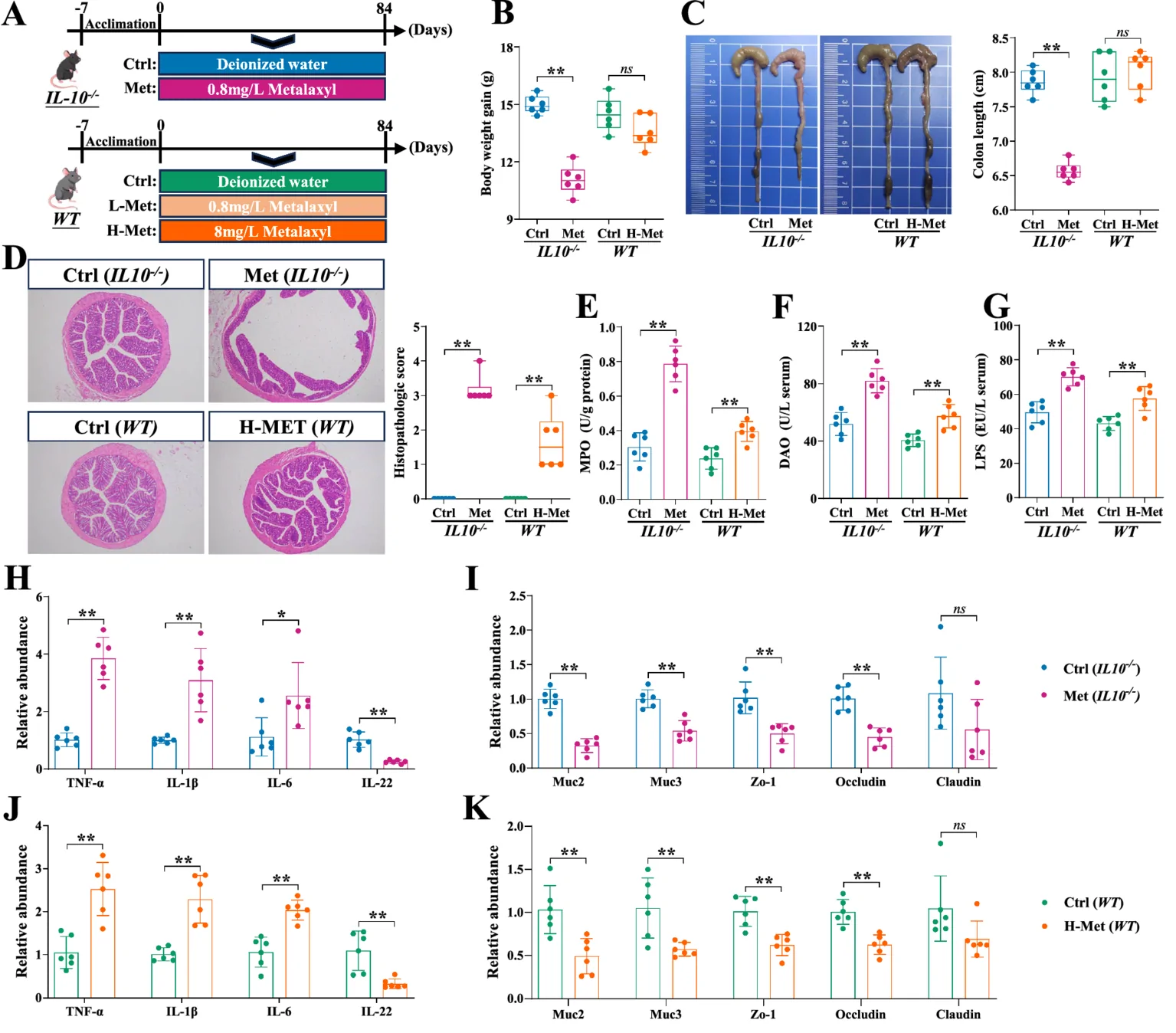
Oral metalaxyl induced colonic inflammation in IL-10 knockout type (*IL-10*^−/−^) mice and wild type (WT) mice. **(A)** Study design for intestinal toxicity testing experiment of metalaxyl. **(B)** Body weight gain. **(C)** Macroscopic pictures of colons and colon length. **(D)** H&E stained colon sections at 40 × and histological scores of colons. **(E)** Concentrations of myeloperoxidase (MPO) in colon. **(F,G)** Concentrations of diamine oxidase (DAO) and lipopolysaccharides (LPS) in serum. **(H,I)** Relative expression of inflammation-related genes and intestinal barrier function-related genes in colon tissue of IL-10^−/−^ mice. **(J,K)** Relative expression of inflammation-related genes and intestinal barrier function-related genes in colon tissue of WT mice. *p < 0.05, **p < 0.01 as compared to Ctrl group, *n =* 6.

To investigate the effect of metalaxyl on the development of IBD, *IL-10*^−/−^ mice (a well-established IBD susceptibility model) were administered with 0.8 mg/L metalaxyl in the drinking water for 12 weeks. Compared with Ctrl group, *IL-10*^−/−^ mice treated with metalaxyl showed limited weight gain (Figure 1B), shorter colon length (Figure 1C), significant infiltration of inflammatory cells and severe atrophy of crypt structure in colon tissue, with significantly increased histological scores (Figure 1D). Besides, the mice in the Met group also exhibited significant increases in the levels of MPO, DAO and LPS (Figures 1E–G), the rise in these biomarkers reflects the presence of intestinal inflammation and the enhancement of intestinal permeability (30–32). Additionally, treatment with metalaxyl led to abnormal expression of a series of genes related to inflammatory responses and barrier structure in the intestine at the transcriptional level, including TNF-α, IL-1β, IL-6, IL-22, Muc2, Muc3, Zo-1, Occludin, and Claudin (Figures 1H–K).

To investigate the effect of metalaxyl on intestinal basal inflammation, wild-type mice were administered 0.8 mg/L and 8 mg/L of metalaxyl. Compared with Ctrl group, treatment with 0.8 mg/L of metalaxyl did not cause significant intestinal inflammation or barrier damage in wild-type mice (Supplementary Figure S1), while treatment with 8 mg/L of metalaxyl led to inflammatory cell infiltration in the colon tissue of wild-type mice, with significantly increasing histological scores (Figure 1D), a significant increase in levels of MPO, DAO and LPS (Figures 1E–G), and abnormal expression of genes related to inflammatory response and barrier structure in the intestine (Figures 1H–K). These data indicated that metalaxyl had a wide range of colitis promoting effects in individuals with different genetic backgrounds.

### 3.2 Metalaxyl exposure disrupted gut microbiota homeostasis of mice

**Figure 2:**
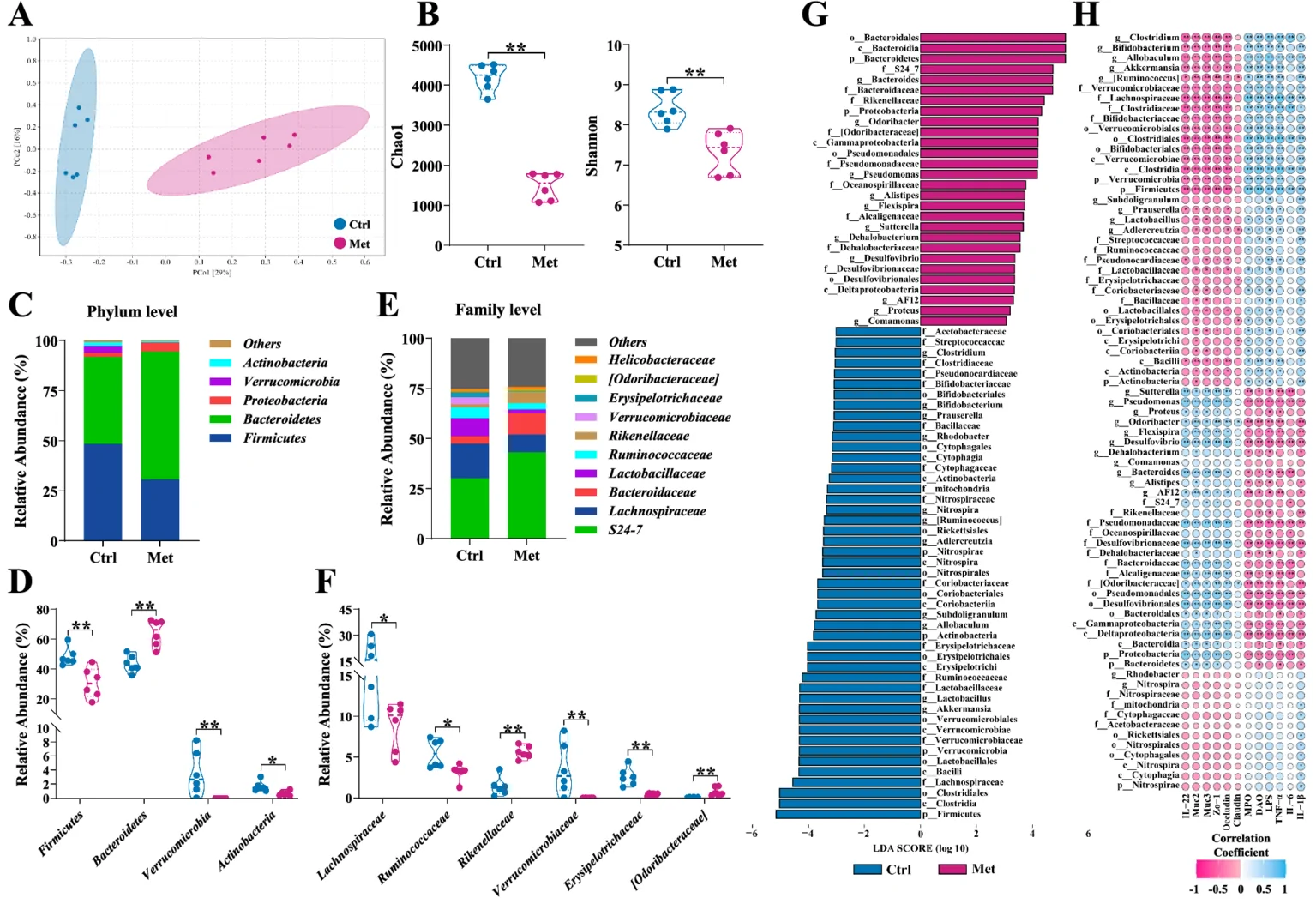
Oral metalaxyl disrupted gut microbiota of *IL-10*^−/−^ mice. **(A)** Weighted UniFrac distance principal coordinate analysis (PCoA); **(B)** alpha-diversity indices; **(C,D)** relative abundance of the gut microbiota at phylum level; **(E,F)** relative abundance of the gut microbiota at family level; **(G)** linear discriminant analysis of microbiome, only features with LDA value of > 3 were shown; **(H)** Spearman correlation analysis of differential microbiota obtained from LDA analysis with intestinal inflammation and barrier-related parameters. *p < 0.05, **p < 0.01 as compared to Ctrl group, *n =* 6.

The gut microbiota plays a crucial role in regulating intestinal immune homeostasis and maintaining mucosal barrier integrity, and its imbalance is considered a key factor in triggering intestinal inflammation and injury (19, 20). Therefore, the changes in the gut microbiota of mice were evaluated to explore the mechanism of metalaxyl in promoting colitis. The 16S rRNA sequencing results of gut microbiota in *IL-10*^−/−^ mice showed that treatment with metalaxyl significantly altered the beta diversity of the gut microbiota, with PCo1 and PCo2 explaining 29 and 16% of the variation (Figure 2A). In addition, exposure to metalaxyl reduced the alpha diversity including the Chao1 richness index and the Shannon diversity index (Figure 2B). Additionally, the species composition of the microbial community also significantly changed. Specifically, at the phylum level, exposure to metalaxyl significantly reduced the relative abundance of *Firmicutes*, *Actinobacteria*, and *Verrucomicrobia*, while significantly increasing the relative abundance of *Bacteroidetes* (Figures 2C,D). At the family level, it led to a significant increase in the relative abundance of *Lachnospiraceae*, *Ruminococcaceae*, and *Erysipelotrichaceae*, and a significant decrease in the relative abundance of *Rikenellaceae* and *Odoribacteraceae* (Figures 2E,F). Moreover, lefse analysis further confirmed significant differences in communitie abundance at various taxonomic levels between Ctrl group and Met group (Figure 2G). Importantly, the abundance changes of these microorganisms after treatment with metalaxyl were highly correlated with parameters of intestinal inflammation and barrier structure damage. Similarly, exposure to metalaxyl also led to significant changes in the beta diversity, alpha diversity, and species composition of the gut microbiota in wild-type mice, and these changes were significantly correlated with the degree of intestinal inflammation and damage (Supplementary Figure S2). These finding revealed that exposure to metalaxyl could significantly disrupt the gut microbiota homeostasis in mice, and changes in some core microbes might play a crucial role in the metalaxyl-induced intestinal inflammation and barrier damage in mice.

### 3.3 Metalaxyl exposure-induced colonic inflammation in mice were transferable through gut microbiota

**Figure 3:**
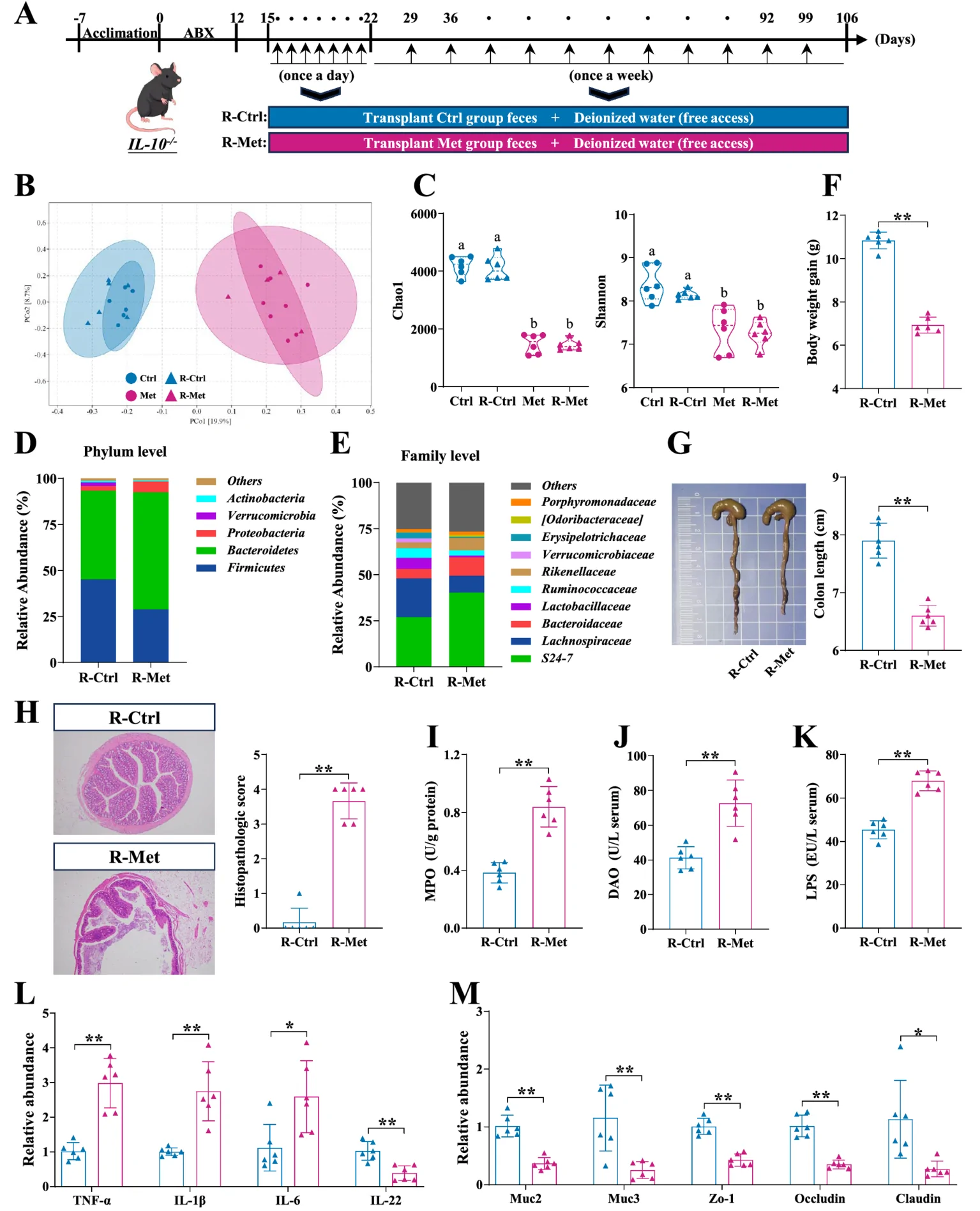
Metalaxyl exposure-induced colonic inflammation in *IL-10*^−/−^ mice was transferable through the gut microbiota. **(A)** Experimental design for fecal microbiota transplantation experiment; **(B)** weighted uniFrac distance principal coordinate analysis (PCoA); **(C)** alpha-diversity indices; **(D)** relative abundance of the gut microbiota at phylum level; **(E)** relative abundance of the gut microbiota at family level; **(F)** body weight gain; **(G)** macroscopic pictures of colons and colon length; **(H)** H&E stained colon sections at 40×, histological scores of colons; **(I)** concentrations of MPO in colon; **(J,K)** concentrations of DAO and LPS in serum; **(L,M)** relative expression of inflammation-related genes and intestinal barrier function-related genes in colon tissue. *p < 0.05, **p < 0.01 as compared to Ctrl group, *n =* 6.

To verify the mediating role of the gut microbiota, mice with pre-cleared gut microbiota were re-colonized with the gut microbiota from either the Ctrl or MET group mice, and then checked if it could transfer intestinal inflammation and structural damage. To begin with, the effectiveness of the fecal microbiota transplantation was evaluated by examining the composition of the gut microbiota in the recipient mice. In *IL-10*^−/−^ mice, the beta and alpha diversity of the gut microbiota did not show significant differences between the intestinal microbiota receptor mice and their corresponding donors, and the differences in beta diversity and alpha diversity between R-Ctrl and R-Met groups still existed in receptor mice (Figures 3B,C). In addition, the differences in species composition between the R-Ctrl group and R-Met group at different taxonomic levels were similar to those observed in donor mice (Figures 3D,E). These results indicated that gut microbiota could be effectively transferred from donor mice to recipient mice.

Next, the intestinal inflammation and damage in the recipient mice were analyzed. In *IL-10*^−/−^ mice, the body weight gain and colon length of the R-MET group were significantly lower than those of the R-Ctrl group (Figures 3F,G). Histological sections of the colon in the R-MET group showed obvious infiltration of inflammatory cells and severe atrophy of the crypt structure, and the histopathological score was significantly higher compared to the R-Ctrl group (Figure 3H). Moreover, the levels of MPO, DAO and LPS were significantly higher in the R-MET group than in the R-Ctrl group (Figures 3I–K). In addition, significant differences in the expression levels of genes related to inflammatory response and barrier structure were still observed between the R-MET and R-Ctrl groups (Figures 3L,M). Similarly, in the wild-type mice, the gut microbiota from the donor mice could be effectively transplanted into the recipient mice, and the phenotypes of intestinal inflammation and barrier structural damage were successfully transferred (Supplementary Figure S3). In summary, these results demonstrated that the intestinal inflammation and barrier structure damage induced by metalaxyl in mice were mediated by gut microbiota.

### 3.4 Metalaxyl exposure disrupted the intestinal metabolic profile of mice

**Figure 4:**
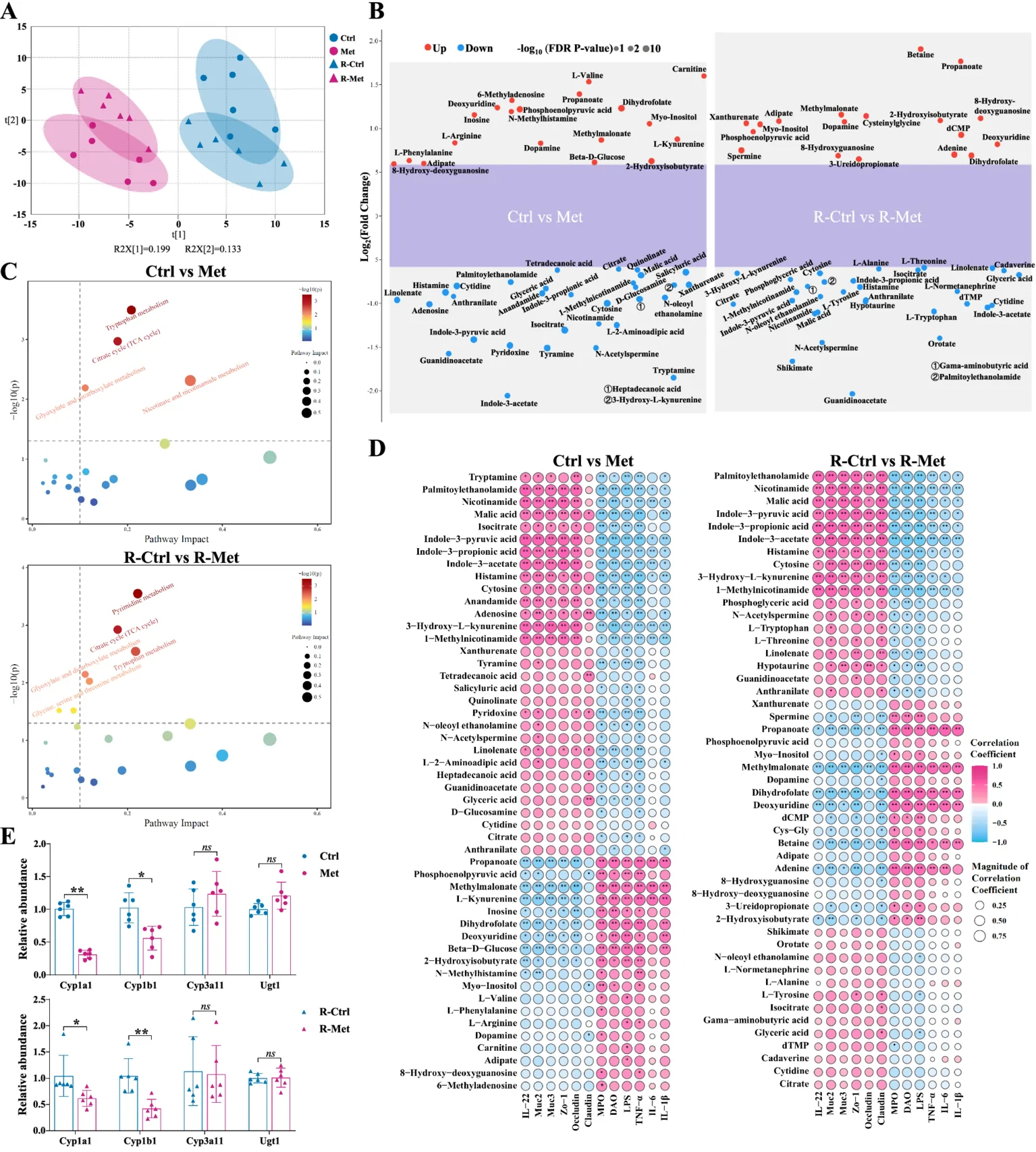
Metalaxyl exposure disrupted the intestinal metabolic profile of *IL-10*^−/−^ mice. **(A)** Principal component analysis (PCA) of all metabolites; **(B)** metabolites with differential abundance; only features with fold change of >1.5 and FDR-*p*-value of < 0.05 were shown; **(C)** pathway analysis of differential metabolites based on KEGG database; **(D)** Pearson correlation analysis of differential metabolites with intestinal inflammation and barrier-related parameters; **(E)** relative expression of AhR downstream target genes *Cyp1a1* and *Cyp1b1* and PXR downstream target genes *Cyp3a11* and *Ugt1*. *p < 0.05, **p < 0.01 as compared to Ctrl group, *n* = 6.

Gut microbial metabolites can act as the output of the combined functions of all microbes to regulate host gut homeostasis (22). To further elucidate the mediating mechanisms of the gut microbiota, the impact of metalaxyl exposure on gut metabolism was explored using a metabolomics approach based on UHPLC–MS/MS. In *IL-10*^*−*/−^ mice, principal component analysis (PCA) revealed a distinct separation in the gut metabolic profiles between the MET group and the Ctrl group (Figure 4A). Based on a threshold of fold change (FC) > 1.5 and false discovery rate (FDR)-corrected *p*-value < 0.05, 50 metabolites were identified with significant changes in abundance following metalaxyl exposure. Specifically, 19 metabolites including carnitine, L-valine, propanoate, 6-methyladenosine, deoxyuridine, dihydrofolate, phosphoenolpyruvic acid, N-methylhistamine, inosine, myo-inositol, methylmalonate, L-arginine, L-kynurenine, dopamine, L-phenylalanine, beta-D-glucose, 2-hydroxyisobutyrate, adipate and 8-hydroxy-deoxyguanosine showed a significant increase in abundance, while 31 metabolites including indole-3-acetate (IAA), tryptamine, guanidinoacetate, N-acetylspermine, tyramine, pyridoxine, indole-3-pyruvic acid, isocitrate, L-2-aminoadipic acid, nicotinamide, cytosine, adenosine, D-glucosamine, heptadecanoic acid, N-oleoyl ethanolamine, linolenate, anthranilate, indole-3-propionic acid (IPA), anandamide, histamine, glyceric acid, xanthurenate, cytidine, 3-hydroxy-L-kynurenine, 1-methylnicotinamide, palmitoylethanolamide, malic acid, salicyluric acid, quinolinate, tetradecanoic acid and citrate exhibited a significant decrease (Figure 4B). Pathway enrichment analysis based on the KEGG database indicated that the pathways of nicotinate and nicotinamide metabolism, tryptophan metabolism, the tricarboxylic acid (TCA) cycle, and glyoxylate and dicarboxylate metabolism were significantly altered following metalaxyl exposure (Figure 4C). Moreover, it was observed that IL-10^−/−^ recipient mice inherited the metabolic characteristics of their corresponding donor mice, and there were significant differences in the gut metabolic profiles between the R-Met group and R-Ctrl group (Figures 4A–C). Similarly, in wild-type mice, changes in the gut microbiota also led to significant alterations in the gut metabolic profile, metabolic pathways such as Nicotinate and nicotinamide metabolism, Alanine, aspartate and glutamate metabolism, the TCA cycle, and Tryptophan metabolism were also significantly affected by metalaxyl exposure (Supplementary Figures S4A–C). Furthermore, Pearson correlation analysis revealed that the abundance changes of metabolites, including 2-hydroxyisobutyrate, deoxyuridine, D-sorbitol, indole-3-acetate, indole-3-pyrubate, nicotinamide and propanoate were significantly correlated with intestinal inflammation and intestinal barrier integrity parameters in all models (Figure 4D and Supplementary Figure S4D). These results indicated that exposure to metalaxyl significantly altered the metabolic profile in mice, and some specific metabolites might play important roles in the gut microbiota-mediated process of metalaxyl-induced intestinal inflammation.

### 3.5 Metalaxyl exposure-induced colonic inflammation in mice was mediated by “tryptophan metabolism-AhR signal transduction” axis

Next, the specific molecular mechanisms by which changes in the metabolic profile drive the occurrence of intestinal inflammation and barrier damage in mice were further investigated. It is worth noting that significant changes were observed in the tryptophan metabolism pathway in all colitis phenotype mice, with the abundance of several tryptophan metabolites including IAA, IPA and indole-3-pyrubate showed a significant negative correlation with the severity of intestinal inflammation (Figure 4 and Supplementary Figure S4). In addition, the combined random forest analysis based on differential microbiota and differential metabolites revealed that tryptophan metabolism-related signatures, including indole-metabolite-producing bacteria (*Lactobacillus* and *Bifidobacterium*) as well as indole metabolites (IAA, IPA) and L-kynurenine, ranked among the top 20 discriminatory features in at least two experimental groups (Supplementary Figure S5). These findings further indicate that microbiota-mediated disturbance of tryptophan metabolism is closely associated with pesticide-triggered intestinal inflammation and intestinal barrier damage. Numerous studies have shown that some tryptophan metabolites could act as ligands to activate AhR or PXR, thereby regulating the production of pro-inflammatory and anti-inflammatory factors, as well as tight junctions and mucin formation in the intestinal mucosa (33, 34). Here, we assessed the activation levels of AhR and PXR. The results showed that the expression levels of the AhR downstream target genes Cyp1a1 and Cyp1b1 were significantly reduced following treatment with metalaxyl, while the expression levels of the PXR downstream target genes remained unchanged (Figure 4E and Supplementary Figure S4E). Therefore, we speculate that the disruption of tryptophan metabolism caused by metalaxyl exposure reduced the activation of AhR, thereby promoting the occurrence of intestinal inflammation and barrier damage in mice.

**Figure 5:**
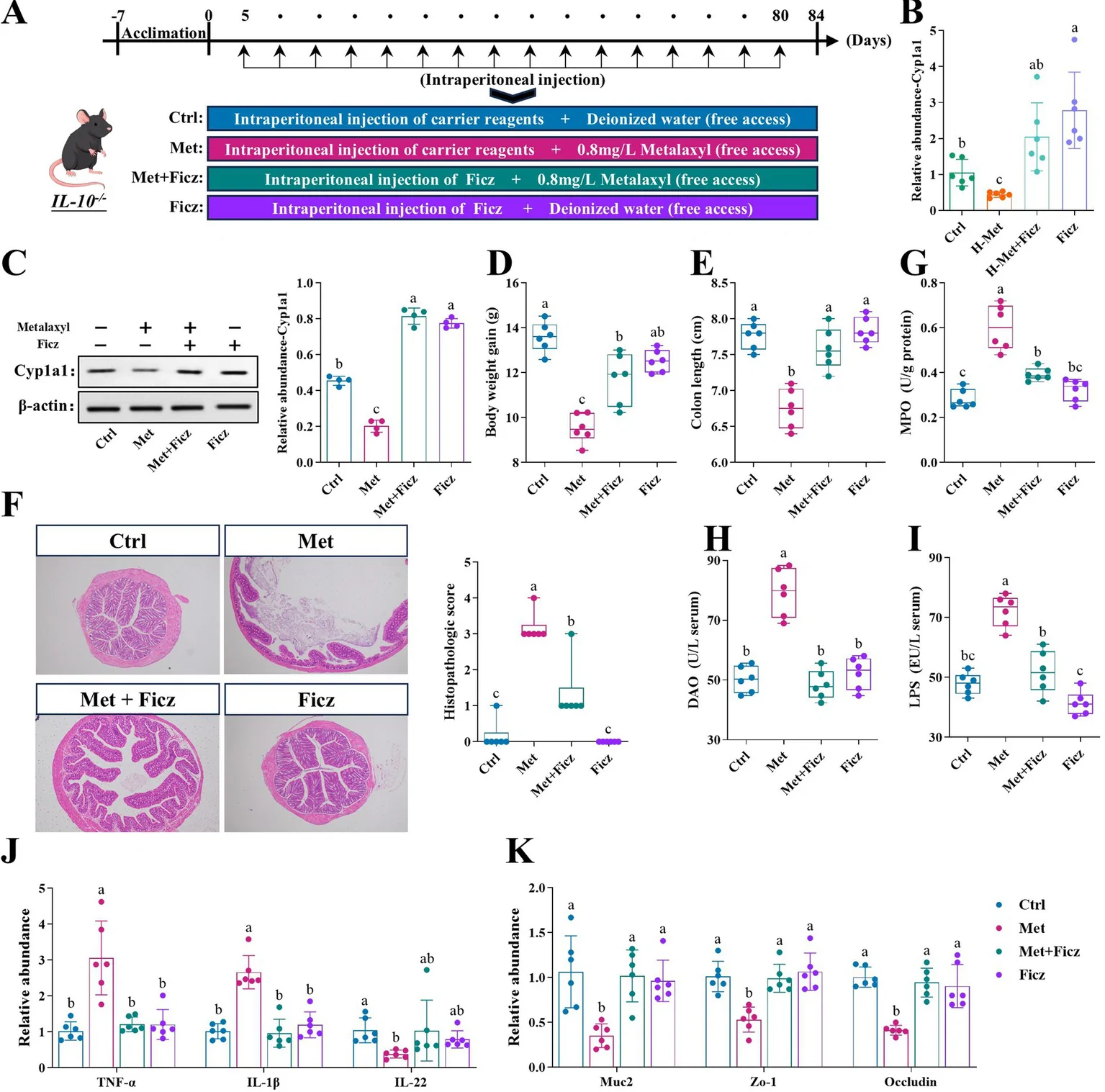
*Metalaxyl exposure-in*duced colonic inflammation in *IL-10*^−/−^ mice were mediated by tryptophan-AhR signaling pathway. **(A)** Study design for AhR activators treatment experiment; **(B)** relative expression levels of AhR target genes Cyp1a1 in colon of IL-10^−/−^ mice; **(C)** protein imprinting analysis and quantitative results of CYP1A1 in AhR activators treatment experiment; **(D)** body weight gain; **(E)** colon length; **(F)** H&E stained colon sections at 40×, Histological scores of colons; **(G)** concentrations of MPO in colon; **(H,I)** Concentrations of DAO and LPS in serum; **(J,K)** relative expression of inflammation-related genes and intestinal barrier function-related genes in colon tissue.

Subsequently, a study was launched to incorporate 6-formylindolo[3,2-b] carbazole, an AhR agonist, during treatment with metalaxyl to validate the role of the “tryptophan metabolism-AhR signal transduction” axis in the progression of intestinal inflammation and barrier damage. As expected, treatment with 6-formylindolo[3,2-b] carbazole reversed the decrease in AhR activation caused by metalaxyl treatment through analyzing the transcription and protein levels of Cyp1a1 (Figures 5B,C). At the same time, in *IL-10*^−/−^ mice, treatment with 6-formylindolo[3,2-b] carbazole significantly improved the weight gain inhibition (Figure 5D), colon length shortening (Figure 5E), intestinal inflammatory cell infiltration, and severe damage to crypt structure caused by metalaxyl (Figure 5F). In addition, the levels of MPO, DAO and LPS in the Met + Ficz group were significantly lower than those in the Met group, with DAO and LPS levels recovering to a level that was not significantly different from the Ctrl group (Figures 5G–I). In addition, unlike the abnormal expression of intestinal inflammatory response and barrier structure related genes caused by treatment with metalaxyl, there was no significant difference in the expression levels of these genes between the Met + Ficz group and the Ctrl group (Figures 5J,K). Similarly, the improvement effect of 6-formylindolo[3,2-b] carbazole was consistently observed in wild-type mice (Supplementary Figure S6). In summary, these results indicated that treatment with 6-formylindolo[3,2-b] carbazole could alleviate or even avoid intestinal inflammation and barrier damage caused by metalaxyl, and revealed that the “tryptophan metabolism-AhR signal transduction” axis was the action pathway through which the gut microbiota mediated metalaxyl-induced colitis.

### 3.6 Prophylactic high tryptophan diet avoided colonic inflammation induced by metalaxyl exposure

**Figure 6:**
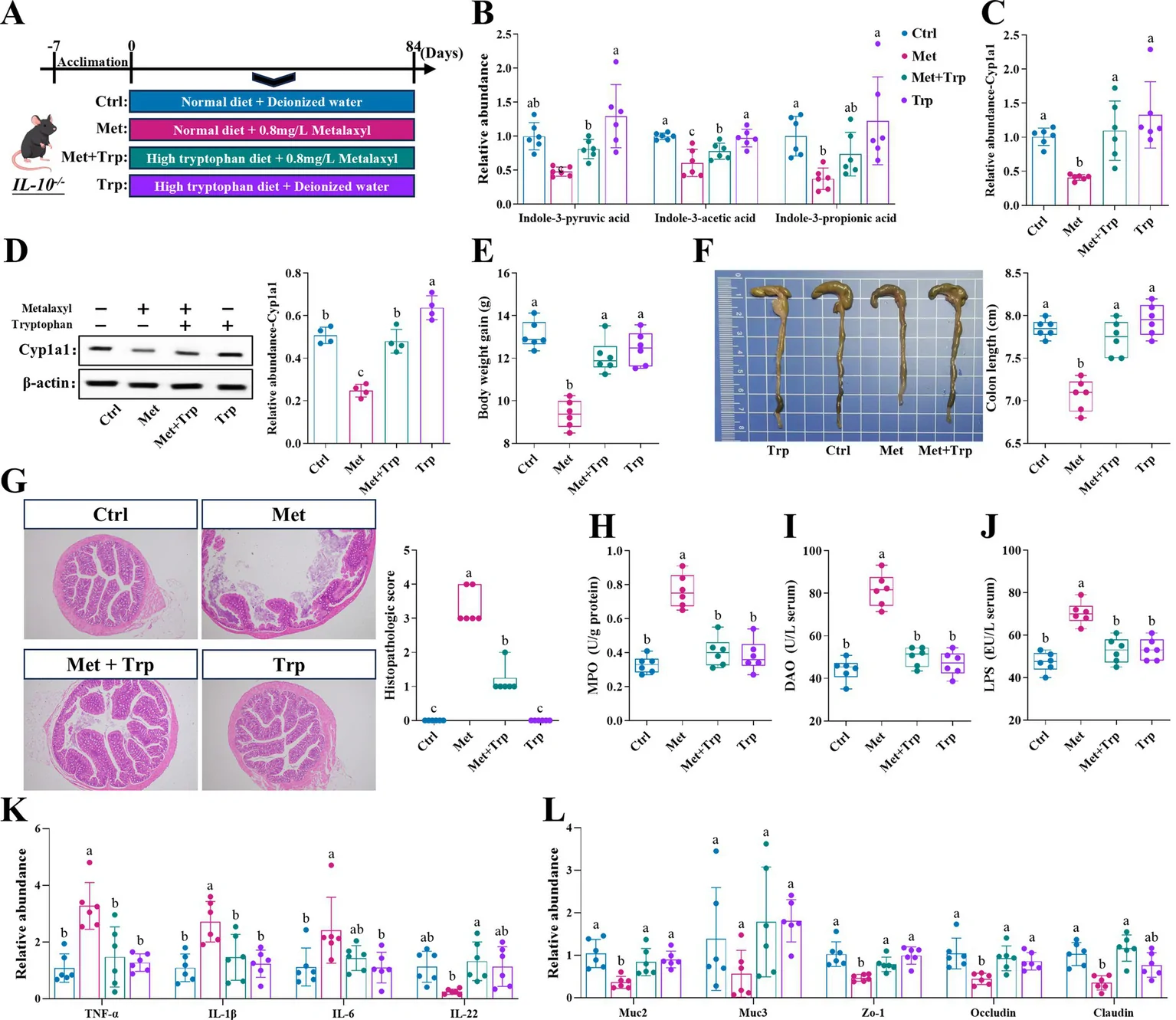
Prophylactic high tryptophan diet avoided colonic inflammation induced by metalaxyl exposure in *IL-10*^−/−^ mice. **(A)** Study design for High Tryptophan Diet treatment experiment. **(B)** Relative abundance of AhR ligands. **(C,D)** Relative expression levels, protein imprinting analysis and quantitative results of Cyp1a1. **(E)** Body weight gain. **(F)** Macroscopic pictures of colons and colon length. **(G)** H&E stained colon sections at 40×, Histological scores of colons. **(H)** Concentrations of MPO in colon. **(I,J)** Concentrations of DAO and LPS in serum. **(K,L)** Relative expression of inflammation-related genes and intestinal barrier function-related genes in colon tissue.

Dietary intervention to increase the availability of AhR ligands is a useful tool to enhance AhR activation (35). Therefore, the potential protective effects of a diet rich in high concentrations of tryptophan on metalaxyl-induced colitis were further investigated. The contents of IAA, IPA and indole-3-pyrubate in the Met + Trp group were significantly higher than those in the Met group, and no significant difference was observed compared with the Ctrl group (Figure 6B), the transcription and protein levels of Cyp1a1 in the Met + Trp group also increased to levels that were not significantly different from those in the Ctrl group (Figures 6C,D), indicating that a tryptophan-rich diet could indeed increase the availability of AhR ligands and enhance AhR activation. At the same time, the high tryptophan diet significantly improved phenotypes such as restricted weight gain (Figure 6E), shortened colon length (Figure 6F), infiltration of intestinal inflammatory cells, and crypt damage caused by metalaxyl (Figure 4G). In addition, unlike the Met group, no significant differences were observed in the levels of MPO, LPS, DAO (Figures 6H–J), as well as in the expression levels of genes related to inflammation and barrier structure (Figures 6K,L) between the Met + Trp group and Ctrl group. Similarly, the improvement effect of a tryptophan-rich diet was consistently observed in wild-type mice (Supplementary Figure S7). These results indicated that a preventive diet rich in high concentrations of tryptophan could effectively counteract metalaxyl-induced colitis.

## 4 Discussion

IBD is a global health challenge, with rising incidence linked to increasing environmental pollutant exposure. Especially, pesticides have also been identified as important risk factors for IBD (2, 36). Metalaxyl is a widely used fungicide that is commonly found in natural environments and has potential hazards (12, 15). However, the adverse effects of metalaxyl on intestinal health are currently unclear, and the mechanism by which fungicides induce intestinal inflammation urgently needs to be further analyzed.

Many studies indicate that both environmental and genetic factors contribute to IBD (37, 38). To comprehensively evaluate the effects of metalaxyl on the intestine of individuals with different genetic backgrounds, IL-10^−/−^ mice with IBD susceptibility and wild-type mice with normal genetic backgrounds were selected for the study. The results showed that metalaxyl could induce severe colitis in *IL-10*^−/−^ mice, and also induced low-grade inflammation in wild type mice, which indicated that the widespread use of metalaxyl might increase the social incidence rate of IBD, especially for individuals with genetic susceptibility to IBD, its adverse effects were more significant. Previous studies have shown that individuals susceptible to IBD and individuals with normal genetic backgrounds have also observed similar toxic effects when exposed to environmental substances such as carboxymethyl cellulose (39), triclocarban (40), and triclosan (41). This phenomenon further supported the synergistic pathogenic effect of environmental pollutants on genetically susceptible populations.

As a key regulator of host health, gut microbiota plays an important role in maintaining intestinal immune homeostasis and mucosal barrier integrity (19, 20). Given the inherent properties of metalaxyl as a fungicide, it might participate in inducing colitis by disrupting the microbial community structure. Microbial analysis of mice showed that metalaxyl significantly reduced microbial diversity and altered the composition of their gut microbiota. Specifically, there was a strong correlation between the abundance of some altered microbiota and the degree of intestinal inflammation. The dysbiosis pattern induced by metalaxyl included a decrease in diversity, a decrease in abundance of *Firmicutes*, *Ruminococcus*, and *Lachnospiraceae*, and an increase in abundance of *Proteobacteria* and *Enterbacteriaceae*, which were similar to the gut microbiota characteristics of typical IBD patients (42–45). These findings suggested that the gut microbiota might be a key mediator of metalaxyl induced colitis. Subsequently, the results of the fecal transplantation experiment showed that after transplanting the microbiota of the donor mice treated with metalaxyl into the recipient mice, the pathological phenotype of gut inflammation was successfully reproduced, further supporting the hypothesis that metalaxyl induced colitis was mediated by gut microbiota.

Significantly, our research results indicated that the occurrence of intestinal inflammation was associated with a majority rather than a few significantly altered microbial species, which also suggested that IBD associated bacteria represented a microbial community rather than the overgrowth of any single bacterial species. This discovery has also been presented in multiple studies (46–48). Accumulating evidence indicated that gut microbiota metabolites, serving as an integrated output of all microbial functional assemblies, directly and comprehensively mediate host gut homeostasis through specific receptors, which has prompted further research on the mechanisms mediated by gut microbiota. Metabolomics analysis of cecal contents showed significant changes in the metabolic profile of cecal contents in the group treated with metalaxyl. More importantly, significant changes in the tryptophan metabolic pathway were observed in all mice with colitis phenotype. A series of derivatives produced by tryptophan metabolism from known microbial sources are important endogenous ligands of AhR, Activation of AhR can regulate the production of pro-inflammatory and anti-inflammatory factors, as well as the formation of tight junctions and mucins in the intestinal mucosa. Our results showed that the abundance of key AhR ligands, including IAA, IPA, and indole-3-pyrubate significantly decreased after exposure to metalaxyl, and this decrease was highly correlated with the occurrence of intestinal inflammation. Therefore, we hypothesized that reduced levels of tryptophan-derived metabolites diminished bioavailability of AhR ligand, leading to impaired AhR activation, which ultimately underlies intestinal inflammation and barrier dysfunction. Subsequently, intervention experiments with AhR agonists confirmed that it could significantly alleviate intestinal inflammation and barrier damage induced by metalaxyl by activating the AhR pathway, providing direct evidence for this mechanism.

Scientific data showed that IBD patients were affected by genetic factors and often had a decreased ability to produce bacterial AhR ligands (49–51). Moreover, as our research confirms, exposure to environmental pollutants might further exacerbate the shortage of these AhR ligands, which undoubtedly increased the incidence and severity of inflammatory bowel disease. However, traditional therapies of IBD have limited efficacy and fail to work for over half of the patients (52). In recent years, diet strategies based on gut microbiota have shown promise, yet more experimental evidence is still needed. We demonstrated that a tryptophan-rich diet effectively elevates levels of protective tryptophan-derived metabolites in the gut, significantly alleviating metalaxyl-induced intestinal inflammation and restoring barrier dysfunction. This study only conducted preventive intervention trials in mouse models, lacking support from gradient dose screening and human clinical trial data, resulting in certain species differences and application limitations; However, this dietary intervention method is safe, non-toxic, and has high compliance, and has the potential to be implemented as a daily supplementary dietary therapy for inflammatory bowel disease in clinical practice. This indicate that the AHR pathway is a promising therapeutic target for intestinal inflammatory diseases, and in the future, a tryptophan-rich diet or a combination with other therapies can be adopted to assist in the prevention and treatment of IBD.

In summary, this study confirmed that metalaxyl induced intestinal inflammation through the “gut microbiota-tryptophan metabolism-AhR signal transduction” axis. The elucidation of this mechanism not only provided evidence linking pesticide exposure, gut microbiota, and IBD progression, but also highlighted the important potential of the AhR pathway as a therapeutic target for intestinal inflammatory diseases.

## 5 Conclusion

This study confirmed that metalaxyl exposure induced low-grade colonic inflammation in wild-type mice and triggered severe colitis in IBD-susceptible mice. In-depth mechanistic investigations revealed that metalaxyl drives intestinal barrier damage and inflammatory cascades via the axis of “gut dysbiosis—impaired tryptophan metabolism—suppressed AhR signaling.” Functional experiments verified that dietary tryptophan supplementation effectively elevates intestinal levels of tryptophan metabolites and rescues AhR signaling activity, thereby markedly alleviating metalaxyl-evoked intestinal inflammation and barrier injury. In summary, these findings not only identify the gut microbiota as a pivotal mediator of environmental pollutant-induced IBD, but also uncover the microbiota-dependent AhR signaling pathway as a core therapeutic target for IBD treatment. High-tryptophan diets exert prominent protective effects in ameliorating environmentally derived inflammatory bowel disease.

## Data Availability

The original contributions presented in the study are publicly available. This data can be found here: https://www.ncbi.nlm.nih.gov/sra/PRJNA1509751.
