# Supplementary material for: Dietary tryptophan ameliorates metalaxyl-induced colitis by restoring microbial tryptophan metabolism and aryl hydrocarbon receptor activation
Source: Front Nutr. 2026 Aug 20;13:1930000. doi: 10.3389/fnut.2026.1930000 (PMC13538018; doi:10.3389/fnut.2026.1930000)
Supplement: Supplementary file 1 [file Table_1.docx]

**Dietary tryptophan ameliorates metalaxyl-induced colitis by restoring microbial tryptophan metabolism and aryl hydrocarbon receptor activation**

Wei Sun^a,b,*^, Yu Wang^b^, Xin zhang^a^, Yifan Yue^b^, Xiaoxuan Sun^b^, Jiyan Miao^b^, Shihang Han^b^, Zhiqiang Zhou^b^, Xiuguo Wang^a^, Wentao Zhu^b,*^.

^a^ State Tobacco Monopoly Administration (China National Tobacco Corporation), Key Laboratory of Tobacco Pest Monitoring Controlling & Integrated Management，Tobacco Research Institute of Chinese Academy of Agricultural Sciences, Qingdao, 266101, China.

^b^ Innovation Center of Pesticide Research, Department of Applied Chemistry, College of Science, China Agricultural University, Beijing 100193, China.

Corresponding author:

* Wei Sun: Tobacco Research Institute of Chinese Academy of Agricultural Sciences, Qingdao, 266101, China.

E-mail address: sunwei05@caas.cn

*Wentao Zhu: Yuanmingyuan west road 2, Beijing, 100193, China.

E-mail address: wentaozhu@cau.edu.cn.

**Table of contents**

**Fig S1.** Oral metalaxyl (0.8mg/L) did not affect the intestinal tract of wild-type mice.

**Fig.S2.** Oral metalaxyl disrupted gut microbiota of WT mice.

**Fig.S3.** Metalaxyl exposure-induced colonic inflammation in WT mice was transferable through the gut microbiota.

**Fig.S4.** Metalaxyl exposure disrupted the intestinal metabolic profile of WT mice.

**Fig.S5.** Metalaxyl exposure-induced colonic inflammation in WT mice were mediated by tryptophan-AhR signaling pathway.

**Fig.S6.** Prophylactic high tryptophan diet avoided colonic inflammation induced by metalaxyl exposure in WT mice.

**Table S1.** Sequences of primers of related genus in qPCR.

**Table S2.** LC-MS/MS qualitative and quantitative ions of metabolites in cecum.

**Fig S1. Oral metalaxyl (0.8mg/L) did not affect the intestinal tract of wild-type mice.** (A) Body weight gain; (B) Macroscopic pictures of colons and colon length; (C) H&E stained colon sections at 40× and histological scores of colons; (D) Concentrations of myeloperoxidase (MPO) in colon; (E-F) Concentrations of diamine oxidase (DAO) and lipopolysaccharides (LPS) in serum; (G-H) Relative expression of inflammation-related genes and intestinal barrier function-related genes in colon tissue of WT mice. n = 6.

**Fig.S2. Oral metalaxyl disrupted gut microbiota of WT mice.** (A) Weighted unifrac distance principal coordinate analysis (PCoA); (B) Alpha-diversity indices; (C-D) Relative abundance of the gut microbiota at phylum level; (E-F) Relative abundance of the gut microbiota at family level; (E) Linear Discriminant Analysis of microbiome, only features with LDA value of ＞3 were shown; (F) Spearman correlation analysis of differential microbiota obtained from LDA analysis with intestinal inflammation and barrier related parameters. *P < 0.05, **P < 0.01 as compared to Ctrl group, n = 6.

**Fig.S3. Metalaxyl exposure-induced colonic inflammation in WT mice was transferable through the gut microbiota.** (A) Weighted unifrac distance principal coordinate analysis (PCoA); (B) Alpha-diversity indices; (C) Relative abundance of the gut microbiota at phylum level; (D) Relative abundance of the gut microbiota at family level; (E) Body weight gain; (F) Macroscopic pictures of colons and colon length; (G) H&E stained colon sections at 40×; (H) Histological scores of colons; (I) Concentrations of MPO in colon; (J-K) Concentrations of DAO and LPS in serum; (L-M) Relative expression of inflammation-related genes and intestinal barrier function-related genes in colon tissue. *P < 0.05, **P < 0.01 as compared to Ctrl group, n = 6.

**Fig.S4. Metalaxyl exposure disrupted the intestinal metabolic profile of WT mice.** (A) Principal component analysis (PCA) of all metabolites; (B) Metabolites with differential abundance, only features with fold change of ＞1.5 and FDR-p value of ＜0.05 were shown; (C) Pathway analysis of differential metabolites based on KEGG database; (D) Pearson correlation analysis of differential metabolites with intestinal inflammation and barrier related parameters. (E) Relative expression of AHR downstream target genes Cyp1a1 and Cyp1b1 and PXR downstream target genes Cyp3a11 and Ugt1. *P < 0.05, **P < 0.01 as compared to Ctrl group, n = 6.

**Fig.S5. Metalaxyl exposure-induced colonic inflammation in WT mice were mediated by tryptophan-AhR signaling pathway.** (A-B) Relative expression levels of AhR target genes CYP1A1 and CYP1B1 in colon of WT mice and WT mice receiving microbiota transplantation; (C-D) Relative expression levels, protein imprinting analysis and quantitative results of CYP1A1 in AhR activators treatment experiment; (E) Body weight gain; (F) Colon length; (G) H&E stained colon sections at 40×; (H) Histological scores of colons; (I) Concentrations of MPO in colon; (J-K) Concentrations of DAO and LPS in serum; (L-M) Relative expression of inflammation-related genes and intestinal barrier function-related genes in colon tissue.

**Fig.S6. Prophylactic high tryptophan diet avoided colonic inflammation induced by metalaxyl exposure in WT mice.** (A) Relative abundance of Ahr ligands; (B-C) Relative expression levels, protein imprinting analysis and quantitative results of CYP1A1; (D) Body weight gain; (E) Macroscopic pictures of colons and colon length; (F) H&E stained colon sections at 40×; (G) Histological scores of colons; (H) Concentrations of MPO in colon; (I-J) Concentrations of DAO and LPS in serum; (K-L) Relative expression of inflammation-related genes and intestinal barrier function-related genes in colon tissue.

**Table S1. Sequences of primers of related genus in qPCR**.

| **Gene** | **Sequences of forward primers** | **Sequences of Reverse primers** |
| --- | --- | --- |
| Muc2 | CTACCACCATTACCACCACTAC | GTCTCTCGATCACCACCATTT |
| Muc3 | TGTTCAGCTTTACTGTGTTTCAA | TTGCATGTCTCCTCAGGATT |
| Zo-1 | GGGGCCTACACTGATCAAGA | TGGAGATGAGGCTTCTGCTT |
| Occludin | ACGGACCCTGACCACTATGA | TCAGCAGCAGCCATGTACTC |
| Claudin | GCCATCTACGAGGGACTGTG | CCCCAGCAGGATGCCAATTA |
| TNF-α | CCCTCACACTCACAAACCAC | ACAAGGTACAACCCATCGGC |
| IL-1β | GCAACTGTTCCTGAACTCAACT | ATCTTTTGGGGTCCGTCAACT |
| IL-6 | AGCCAGAGTCCTTCAGAGAGA | GCCACTCCTTCTGTGACTCC |
| IL-22 | TGCAAGCTTGAGGTGTCCAA | AGCCGGACATCTGTGTTGTT |
| AhR | GAGGTGGGTCCAGTCCAATG | GTCAGTGGTCTCTGAGTGGC |
| CYP1A1 | ACATTGTGCCTGCCTCCTAC | GTAGGGTGAACAGAGGTGCC |
| CYP1B1 | CAAGTGGCCTAACCCAGAGG | TCATCGTGGTGTGCCATCC |
| PXR | TCAAGGATTTCCGGCTGCG | GTAGGTTGACACATCGGCCA |
| CYP3A11 | GTGCTCCTAGCAATCAGCTT | CAGTGCCTAAAAATGGCAGAGG |
| Ugt1 | GGACACGGGACTATGTGTGC | CTGCCCTCCATGGGTAACAC |
| GAPDH | GTGGACCTCATGGCCTACAT | TGTGAGGGAGATGCTCAGTG |
| 16s | (338F) ACTCCTACGGGAGGCGCAGCA | (806R) GGACTACHVGGGTWTCTAAT |

**Table S2. LC-MS/MS qualitative and quantitative ions of metabolites in cecum.**

| **No** | **Metabolites** | **Qualitative ion** | **Quantitative ion** | **Declustering potential** | **Collision energy** | **Mode** |
| --- | --- | --- | --- | --- | --- | --- |
| 1 | Trimethylamine | 60.000 | 44.000 | 150 | 30 | + |
| 2 | L-Glycine | 76.000 | 30.100 | 30 | 16 | + |
| 3 | Trimethylamine-N-oxide | 76.100 | 58.000 | 75 | 28 | + |
| 4 | Putrescine | 89.000 | 72.000 | 60 | 15 | + |
| 5 | Sarcosine | 89.900 | 44.000 | 50 | 18 | + |
| 6 | L-Alanine | 90.000 | 44.000 | 60 | 20 | + |
| 7 | Glycerol | 93.000 | 57.000 | 100 | 20 | + |
| 8 | Cadaverine | 103.000 | 86.000 | 100 | 12 | + |
| 9 | Choline | 104.100 | 60.000 | 150 | 25 | + |
| 10 | Dimethylglycine | 104.100 | 58.000 | 50 | 20 | + |
| 11 | 3-Aminoisobutyric acid | 104.100 | 86.000 | 100 | 20 | + |
| 12 | γ-Aminobutyric acid | 104.100 | 87.000 | 26 | 13 | + |
| 13 | L-Serine | 106.000 | 60.000 | 50 | 17 | + |
| 14 | Hypotaurine | 109.900 | 65.200 | 35 | 13 | + |
| 15 | Histamine | 112.000 | 95.000 | 50 | 20 | + |
| 16 | Cytosine | 112.100 | 95.000 | 50 | 25 | + |
| 17 | Creatinine | 114.100 | 44.000 | 75 | 35 | + |
| 18 | L-Proline | 116.100 | 70.000 | 55 | 25 | + |
| 19 | 5-Aminopentanoic acid | 118.000 | 55.000 | 50 | 25 | + |
| 20 | Betaine | 118.000 | 58.000 | 175 | 20 | + |
| 21 | L-Valine | 118.100 | 72.000 | 60 | 30 | + |
| 22 | L-Norvaline | 118.100 | 72.000 | 60 | 20 | + |
| 23 | Indole | 118.100 | 91.000 | 150 | 25 | + |
| 24 | L-Homoserine | 120.100 | 74.000 | 50 | 15 | + |
| 25 | L-Threonine | 120.100 | 74.000 | 50 | 15 | + |
| 26 | L-Cysteine | 122.000 | 59.000 | 60 | 20 | + |
| 27 | Nicotinamide | 123.000 | 80.000 | 75 | 25 | + |
| 28 | Nicotinate | 124.100 | 78.000 | 135 | 25 | + |
| 29 | Taurine | 126.000 | 108.000 | 75 | 15 | + |
| 30 | N-Methylhistamine | 126.000 | 109.000 | 50 | 25 | + |
| 31 | 1 -Piperideine-6-carboxylic acid | 128.000 | 82.000 | 101 | 17 | + |
| 32 | 5-Oxoproline | 130.000 | 83.400 | 75 | 15 | + |
| 33 | L-Pipecolate | 130.000 | 84.000 | 75 | 25 | + |
| 34 | Agmatine | 131.000 | 72.000 | 75 | 23 | + |
| 35 | N-Acetylputrescine | 131.100 | 114.000 | 50 | 15 | + |
| 36 | Ethyl 4-aminobutyrate | 131.700 | 87.000 | 50 | 19 | + |
| 37 | Creatine | 132.100 | 90.000 | 60 | 20 | + |
| 38 | Hydroxyproline | 132.100 | 86.200 | 75 | 20 | + |
| 39 | 5-Aminolevulinate | 132.100 | 86.200 | 75 | 20 | + |
| 40 | L-Isoleucine | 132.100 | 86.000 | 40 | 15 | + |
| 41 | L-Leucine | 132.100 | 86.000 | 100 | 15 | + |
| 42 | L-Ornithine | 133.100 | 70.000 | 30 | 15 | + |
| 43 | L-Asparagine | 133.100 | 74.000 | 75 | 20 | + |
| 44 | L-Aspartate | 134.100 | 74.000 | 75 | 20 | + |
| 45 | L-Homocysteine | 136.000 | 90.000 | 50 | 15 | + |
| 46 | 1-Methylnicotinamide | 137.000 | 94.100 | 70 | 27 | + |
| 47 | Tyramine | 138.100 | 77.000 | 65 | 33 | + |
| 48 | Anthranilate | 138.100 | 120.000 | 35 | 20 | + |
| 49 | 4-Acetamidobutanoate | 146.000 | 86.100 | 80 | 23 | + |
| 50 | Spermidine | 146.100 | 72.000 | 125 | 20 | + |
| 51 | Acetylcholine | 146.100 | 87.000 | 100 | 25 | + |
| 52 | L-Glutamine | 147.100 | 84.000 | 50 | 25 | + |
| 53 | L-Lysine | 147.101 | 84.000 | 75 | 25 | + |
| 54 | L-Glutamate | 148.100 | 84.000 | 55 | 20 | + |
| 55 | L-Methionine | 150.100 | 61.000 | 55 | 30 | + |
| 56 | Cysteamine | 153.000 | 108.000 | 50 | 30 | + |
| 57 | 3-Hydroxyanthranilate | 154.000 | 80.000 | 50 | 35 | + |
| 58 | L-Histidine | 156.100 | 110.000 | 40 | 20 | + |
| 59 | L-2-Aminoadipic acid | 162.050 | 98.000 | 46 | 21 | + |
| 60 | Tryptamine | 161.100 | 144.000 | 50 | 20 | + |
| 61 | Carnitine | 162.100 | 85.000 | 75 | 30 | + |
| 62 | L-Phenylalanine | 166.100 | 120.000 | 60 | 20 | + |
| 63 | Quinolinic acid | 168.000 | 149.900 | 41 | 15 | + |
| 64 | N-Methyl-L-histidine | 170.000 | 96.000 | 75 | 30 | + |
| 65 | Pyridoxine | 170.100 | 152.100 | 45 | 20 | + |
| 66 | L-Arginine | 175.100 | 70.000 | 60 | 30 | + |
| 67 | Indole-3-acetate | 176.100 | 130.000 | 50 | 30 | + |
| 68 | Serotonin | 177.000 | 160.000 | 50 | 10 | + |
| 69 | Cys-Gly | 179.100 | 76.000 | 50 | 35 | + |
| 70 | D-Glucosamine | 180.100 | 162.000 | 50 | 15 | + |
| 71 | D-Mannose | 181.000 | 99.000 | 150 | 25 | + |
| 72 | L-Tyrosine | 182.100 | 136.000 | 75 | 20 | + |
| 73 | L-Adrenaline | 184.000 | 166.200 | 150 | 25 | + |
| 74 | L-Normetanephrine | 184.100 | 166.200 | 150 | 25 | + |
| 75 | N8-Acetylspermidine | 188.000 | 114.100 | 60 | 23 | + |
| 76 | Kynurenic acid | 190.000 | 144.000 | 50 | 30 | + |
| 77 | Indole-3-propionic acid | 190.000 | 130.000 | 50 | 20 | + |
| 78 | 5-Hydroxy-indole-acetate | 192.100 | 146.000 | 50 | 25 | + |
| 79 | Salicyluric acid | 196.100 | 168.000 | 40 | 15 | + |
| 80 | Spermine | 203.100 | 129.000 | 150 | 18 | + |
| 81 | O-Acetylcarnitine | 204.100 | 85.000 | 100 | 25 | + |
| 82 | Indole-3-Pyruvate | 204.100 | 158.000 | 50 | 20 | + |
| 83 | L-Tryptophan | 205.100 | 146.000 | 75 | 25 | + |
| 84 | Indolelactate | 206.000 | 118.000 | 50 | 30 | + |
| 85 | Xanthurenate | 206.000 | 160.000 | 50 | 15 | + |
| 86 | L-Kynurenine | 209.100 | 94.000 | 75 | 20 | + |
| 87 | 5-Hydroxy-L-tryptophan | 221.150 | 204.050 | 50 | 10 | + |
| 88 | 3-Hydroxy-L-kynurenine | 225.100 | 208.050 | 50 | 10 | + |
| 89 | Deoxyuridine | 229.100 | 113.000 | 60 | 20 | + |
| 90 | Melatonin | 233.100 | 174.100 | 50 | 15 | + |
| 91 | L-Cystine | 241.100 | 120.000 | 60 | 25 | + |
| 92 | Cytidine | 244.100 | 112.100 | 60 | 20 | + |
| 93 | N1-Acetylspermine | 245.100 | 100.100 | 30 | 26 | + |
| 94 | Uridine | 245.100 | 113.100 | 60 | 15 | + |
| 95 | Adenosine | 268.100 | 136.100 | 60 | 20 | + |
| 96 | Inosine | 269.100 | 137.100 | 60 | 15 | + |
| 97 | 1-Methyladenosine | 282.100 | 150.100 | 40 | 25 | + |
| 98 | 6-Methyladenosine | 282.160 | 150.100 | 60 | 25 | + |
| 99 | 8-Hydroxy-deoxyguanosine | 284.150 | 168.100 | 60 | 25 | + |
| 100 | Guanosine | 284.150 | 185.200 | 100 | 16 | + |
| 101 | Methylthioadenosine | 298.100 | 136.100 | 20 | 26 | + |
| 102 | 8-hydroxyguanosine | 300.150 | 151.000 | 30 | 15 | + |
| 103 | Palmitoylethanolamide | 300.400 | 61.900 | 27 | 10 | + |
| 104 | N-oleoyl ethanolamine | 326.500 | 61.900 | 27 | 16 | + |
| 105 | Anandamide | 348.200 | 287.300 | 27 | 14 | + |
| 106 | 2-Arachidonoylglycerol | 379.200 | 269.200 | 44 | 22 | + |
| 107 | S-Adenosylmethionine | 399.100 | 250.100 | 100 | 21 | + |
| 108 | Bilirubin | 585.300 | 299.200 | 80 | 29 | + |
| 109 | Malondialdehyde | 71.000 | 41.000 | -60 | -25 | － |
| 110 | Propanoate | 73.000 | 55.000 | -40 | -20 | － |
| 111 | Pyruvate | 87.000 | 43.000 | -65 | -10 | － |
| 112 | 2-Methylpropanoate | 87.000 | 42.900 | -65 | -20 | － |
| 113 | L-Lactate | 89.000 | 43.000 | -100 | -15 | － |
| 114 | Glyceraldehyde | 89.000 | 59.000 | -40 | -10 | － |
| 115 | Oxalate | 89.000 | 42.900 | -50 | -17 | － |
| 116 | 3-Methylbutanoic acid | 101.000 | 84.000 | -70 | -30 | － |
| 117 | 4-Aminobutanoate | 102.100 | 84.000 | -50 | -14 | － |
| 118 | Malonate | 103.000 | 41.000 | -75 | -32 | － |
| 119 | 3-Hydroxybutyric acid | 103.000 | 59.000 | -60 | -15 | － |
| 120 | Glyceric acid | 105.000 | 75.000 | -50 | -15 | － |
| 121 | Uracil | 111.000 | 42.000 | -50 | -30 | － |
| 122 | Fumarate | 115.000 | 71.000 | -60 | -15 | － |
| 123 | 2-Oxoisovalerate | 115.100 | 87.100 | -80 | -11 | － |
| 124 | Guanidinoacetate | 116.000 | 74.000 | -60 | -15 | － |
| 125 | 2-Hydroxyisovaleric acid | 117.000 | 71.000 | -60 | -18 | － |
| 126 | Succinate | 117.000 | 73.000 | -60 | -17 | － |
| 127 | Methylmalonate | 117.000 | 73.000 | -40 | -13 | － |
| 128 | 2-Hydroxyisobutyrate | 117.000 | 85.000 | -75 | -18 | － |
| 129 | 4-Methyl-2-oxopentanoate | 128.700 | 69.000 | -80 | -28 | － |
| 130 | 2-Methylmaleate | 129.000 | 85.000 | -50 | -15 | － |
| 131 | 3-Methyl-2-oxopentanoate | 129.000 | 101.000 | -70 | -15 | － |
| 132 | MethylSuccinate | 131.000 | 69.000 | -60 | -27 | － |
| 133 | 2-Hydroxyisocaproate | 131.000 | 85.000 | -75 | -20 | － |
| 134 | Glutarate | 131.000 | 87.000 | -60 | -15 | － |
| 135 | Oxalacetate | 131.000 | 113.000 | -60 | -15 | － |
| 136 | 3-Ureidopropionate | 131.000 | 115.000 | -125 | -18 | － |
| 137 | Malate | 133.000 | 115.000 | -75 | -15 | － |
| 138 | Adenine | 134.100 | 107.000 | -60 | -25 | － |
| 139 | Hypoxanthine | 135.000 | 92.000 | -70 | -23 | － |
| 140 | Adipate | 144.900 | 83.000 | -60 | -20 | － |
| 141 | α-ketoglutarate | 145.000 | 57.100 | -50 | -16 | － |
| 142 | 2-Oxoglutarate | 145.000 | 101.000 | -60 | -25 | － |
| 143 | 2-Hydroxyglutarate | 147.000 | 129.000 | -60 | -16 | － |
| 144 | Xanthine | 151.000 | 108.000 | -75 | -30 | － |
| 145 | Dopamine | 152.100 | 122.000 | -70 | -20 | － |
| 146 | Orotate | 155.000 | 111.000 | -30 | -18 | － |
| 147 | Phenylpyruvate | 163.000 | 91.000 | -40 | -15 | － |
| 148 | Quinolinate | 166.000 | 78.000 | -50 | -20 | － |
| 149 | Phosphoenolpyruvic acid | 166.900 | 79.000 | -40 | -25 | － |
| 150 | Homogentisate | 167.000 | 123.000 | -60 | -20 | － |
| 151 | Dihydroxyacetone phosphate | 168.900 | 78.800 | -60 | -40 | － |
| 152 | D-Glyceraldehyde 3-phosphate | 168.900 | 97.000 | -60 | -16 | － |
| 153 | sn-Glycerol 3-phosphate | 171.000 | 79.000 | -175 | -35 | － |
| 154 | Shikimate | 173.000 | 93.000 | -60 | -20 | － |
| 155 | Cis-aconitate | 173.000 | 85.000 | -20 | -15 | － |
| 156 | Hippurate | 178.000 | 134.000 | -40 | -17 | － |
| 157 | Myo-Inositol | 179.000 | 87.000 | -100 | -21 | － |
| 158 | β-D-Glucose | 179.000 | 89.000 | -75 | -15 | － |
| 159 | 4-Hydroxyphenylpyruvate | 179.000 | 107.100 | -50 | -13 | － |
| 160 | Homovanillate | 181.000 | 137.000 | -75 | -20 | － |
| 161 | 4-Pyridoxate | 182.100 | 138.000 | -75 | -20 | － |
| 162 | Phosphoglyceric acid | 185.000 | 79.000 | -40 | -20 | － |
| 163 | Azelaic acid | 187.000 | 125.000 | -100 | -20 | － |
| 164 | Citrate | 191.000 | 87.000 | -40 | -25 | － |
| 165 | Isocitrate | 191.000 | 117.000 | -50 | -20 | － |
| 166 | D-Glucuronate | 193.000 | 113.100 | -50 | -17 | － |
| 167 | Pantothenate | 218.100 | 88.000 | -80 | -20 | － |
| 168 | 3-Nitrotyrosine | 225.000 | 163.000 | -125 | -20 | － |
| 169 | Tetradecanoic acid | 227.100 | 209.000 | -150 | -28 | － |
| 170 | D-Ribose 5-phosphate | 229.000 | 97.000 | -50 | -35 | － |
| 171 | Pyridoxal phosphate | 246.000 | 96.900 | -60 | -20 | － |
| 172 | F6P/F1P | 259.000 | 169.000 | -40 | -20 | － |
| 173 | G6P | 259.000 | 199.000 | -40 | -20 | － |
| 174 | G1P | 259.000 | 241.000 | -40 | -20 | － |
| 175 | Heptadecanoic acid | 269.100 | 251.300 | -175 | -32 | － |
| 176 | Linoleate | 277.100 | 259.000 | -70 | -30 | － |
| 177 | Linolenate | 279.100 | 261.000 | -70 | -25 | － |
| 178 | Xanthosine | 283.100 | 151.000 | -75 | -21 | － |
| 179 | L-Argininosuccinate | 289.100 | 271.100 | -50 | -15 | － |
| 180 | Arachidonate | 303.300 | 259.200 | -80 | -20 | － |
| 181 | dCMP | 306.000 | 79.000 | -70 | -50 | － |
| 182 | Glutathione | 306.100 | 143.000 | -125 | -30 | － |
| 183 | dTMP | 321.100 | 79.000 | -60 | -50 | － |
| 184 | CMP | 322.000 | 97.000 | -150 | -40 | － |
| 185 | G16BP | 339.000 | 241.000 | -50 | -25 | － |
| 186 | Lactose | 341.000 | 59.000 | -125 | -45 | － |
| 187 | cGMP | 344.000 | 150.000 | -70 | -40 | － |
| 188 | IMP | 347.000 | 79.000 | -175 | -45 | － |
| 189 | UDP | 403.000 | 79.000 | -70 | -70 | － |
| 190 | Folate | 440.100 | 311.000 | -60 | -35 | － |
| 191 | Dihydrofolate | 442.100 | 366.200 | -40 | -15 | － |
| 192 | Tetrahydrofolate | 444.100 | 385.200 | -60 | -20 | － |
| 193 | 5-Methyltetrahydrofolate | 458.200 | 329.100 | -40 | -35 | － |
| 194 | Glycocholate | 464.300 | 74.000 | -100 | -75 | － |
| 195 | ATP | 505.900 | 159.000 | -90 | -37 | － |
| 196 | Taurocholate | 514.200 | 80.100 | -50 | -140 | － |
| 197 | GTP | 522.000 | 424.000 | -90 | -37 | － |
| 198 | Glutathione disulfide | 611.200 | 306.000 | -90 | -37 | － |
